# Breastfeeding moderates *FTO* related adiposity: a birth cohort study with 30 years of follow-up

**DOI:** 10.1038/s41598-018-20939-4

**Published:** 2018-02-07

**Authors:** Bernardo Lessa Horta, Cesar G. Victora, Giovanny V. A. França, Fernando P. Hartwig, Ken K. Ong, Emanuella de Lucia. Rolfe, Elma I. S. Magalhães, Natalia P. Lima, Fernando C. Barros

**Affiliations:** 1 Postgradute Programme in Epidemiology, Universidade Federal de Pelotas, Pelotas, Rio Grande do Sul Brazil; 2 Secretariat of Health Surveillance, Ministry of Health, Brasilia, Brazil; 3 Medical Research Council (MRC) Epidemiology Unit, Institute of Metabolic Science, University of Cambridge School of Clinical Medicine, Cambridge, England; 4 Universidade Católica de Pelotas, Pelotas, Rio Grande do Sul Brazil

## Abstract

This study assessed the association of breastfeeding with body composition at 30 years, among subjects who have been prospectively followed since birth in a southern Brazilian city. We also evaluated whether breastfeeding moderated the association between the rs9939609 variant in the *FTO* gene and adiposity. At 30 years, total and predominant breastfeeding were positively associated with lean mass index and inversely with visceral fat thickness. Among subjects breastfed for <1 month, all outcomes showed monotonically increasing values with additional copies of the A allele in the *FTO* genotype (rs9939609). Associations among subjects breastfed for one month or longer tended to be in the same direction but showed lower magnitude and were less consistent; for all outcomes. Interactions had p values ≤ 0.05 for body mass index, fat mass index and waist circumference. Even among young adults, breastfeeding moderates the association between the *FTO* variant rs9939609 and body composition.

## Introduction

Breastfeeding has clear short-term benefits, reducing morbidity and mortality from infectious diseases^1,2^ but breastfeeding may also have long-term benefits. For example, breastfeeding is positively associated with performance in intelligence tests in childhood and adulthood^3,4^. Breastfeeding duration is also positively associated with adult earnings likely to be largely mediated by intelligence^5^. Furthermore, breastfeeding could also protect against the development of noncommunicable diseases. A recently published meta-analysis^6^ reported a negative association of breastfeeding with overweight/obesity in childhood and adulthood, and the association was still observed among those studies with more than 1500 participants, that controlled for confounding and with a short-term maternal recall of breastfeeding [pooled odds ratio: 0.87 (95% confidence interval: 0.76; 0.99)]. Breastfeeding was also associated with a lower odds of type-2 diabetes [pooled odds ratio: 0.65 (95% confidence interval: 0.49; 0.86)]. Recently, Lewandowski *et al*.^7^ reported that preterm offspring who had been exclusively breastfed had increased left and right ventricular end-diastolic volume index compared to those who had not been breastfed.

Concerning the association of breastfeeding with obesity, one of the possible mechanisms is that breastfeeding would be associated with better development of self-regulation of energy intake and satiety responsiveness^8^. Some studies have evaluated whether breastfeeding would moderate the effect of genetic variants in the *FTO* gene, which are associated with obesity, via satiety and intake of energy dense-foods^9–11^. It was observed in two Greek cohorts that breastfeeding moderated the association of rs17817449 and rs9939609 single nucleotide polymorphism (SNP) in *FTO* with adiposity, but these findings were not replicated in the ALSPAC cohort^11^. In the Raine cohort-Australia, Abarin *et al*.^12^ also observed that breastfeeding attenuated the association of rs9939609 with body mass index in childhood and adolescence. These findings reinforce the hypothesis that the programing of satiety control is one of the possible mechanisms for the association between breastfeeding and obesity.

Few studies have evaluated long-term associations between breastfeeding and specific fat measurements and body fat distribution. Toschke *et al*.^13^ reported that children who had been breastfed for 6 or more months had the lowest odds of total fat mass in the top decile at age 9–10 years. Other studies have also reported that breastfeeding was associated with lower body fat percentages or body fat distribution^14–18^. On the other hand, some studies have not detected such association^19–22^. In the Promotion of Breastfeeding Intervention Trial, body fat percentage at 16 years was similar among subjects whether or not they were randomly allocated to receive or not a breastfeeding promotion intervention, whereas the prevalence of overweight/obesity was higher in the intervention arm^23^.

Most of the studies on the long-term consequences of breastfeeding have been carried out in high-income countries, where socioeconomic status is positively associated with breastfeeding duration^24^. Therefore, it has been suggested that the apparent programming effect of breastfeeding may be due to residual confounding. Given that such socioeconomic patterning of breastfeeding duration is not present in the 1982 Pelotas (Southern Brazil) birth cohort^25^, findings from this cohort would not be susceptible to residual confounding from socioeconomic status.

This study was aimed at assessing the association of breastfeeding duration with body composition at 30 years of age. We also evaluated whether breastfeeding moderated the association between the rs9939609 variant in the FTO gene and adiposity.

## Results

In the 2012–13 visit, we interviewed 3701 participants, who added to the 325 known to have died, represented a follow-up rate of 68.1%. Information on breastfeeding duration and at least one of the outcomes was available for 3461 subjects. The *FTO* SNP rs9939609 was in Hardy-Weinberg equilibrium (p = 0.69). The frequency distributions for genotype TT, TA, and AA were 33.6%, 48.4% and 18.0%, respectively. Table 1 shows selected characteristics of the cohort, including information on confounding variables. Mean birthweight was 3227 g, and only 13.7% of the mothers had completed secondary school (12 or more years). Concerning infant feeding, 21.3% of the individuals were breastfed for less than 1 month, whereas 30.0% breastfed for 6 or more months. Duration of predominant breastfeeding was short, and only about four of every ten subjects were predominantly breastfed for three months or more. At the age of 30 years, mean body mass index was 26.8 kg/m^2^.

**Table 1:** Characteristics of participants included in the present analysis.

Characteristic	Total, No (%) (N=3461)*
**Variables measured at birth**	
Maternal schooling, y	
0–4	1108 (32.1)
5–8	1497 (43.3)
9–11	377 (10.9)
≥12	475 (13.7)
Birthweight, mean (SD), g	3227 (528)
**Gestational age, w**	
<37	156 (5.6)
≥37	2632 (94.4)
Variables measured during childhood	
Duration of breastfeeding, m	
<1	736 (21.3)
1–2.9	890 (25.7)
3–5.9	795 (23.0)
≥6	1040 (30.0)
Duration of predominant breastfeeding, m	
<1	889 (26.5)
1 1.9	458 (13.7)
2–2.9	678 (20.2)
≥3	1325 (39.6)
**Variables measured at 30 years of age**	
Body mass index, mean (SD), kg/m^2^	26.8 (5.5)
Fat mass index, mean (SD), kg/m^2^	8.77 (4.34)
Lean mass index, mean (SD), kg/m^2^	16.7 (2.67)
Waist circumference, mean (SD), cm	84.9 (12.7)
Visceral fat thickness, mean (SD), cm	5.92 (2.11)
Subcutaneous abdominal fat thickness, mean (SD), cm	2.24 (1.11)

*The total of some variables does not sum to 3461 because of missing data.

With respect to the association of breastfeeding duration with the confounders, we did not observe any clear pattern of association with socioeconomic variables (family income and maternal schooling). The proportion of subjects who were breastfed for at least 6 months was higher among those in the extreme categories of family income and maternal schooling. Birthweight and prepregnancy maternal body mass index were positively associated with duration of breastfeeding. On the other hand, offspring of mothers who smoked in the pregnancy were less likely of being breastfed at 6 months (Table 2).

**Table 2:** Distribution of confounding variables according to duration of breastfeeding.

	Duration of breastfeeding (months)			
	<1	1–2.9	3–5.9	≥6
Family income at birth (quintiles)				
1^st^	23.1%	26.4%	19.6%	30.9%
2^nd^	19.3%	26.1%	21.5%	33.2%
3^rd^	24.9%	25.7%	21.9%	27.4%
4^th^	22.6%	27.2%	24.5%	25.7%
5^th^	16.4%	22.6%	27.7%	33.4%
Maternal schooling (years)				
0–4	21.9%	26.3%	19.5%	32.3%
5–8	23.4%	26.3%	23.3%	27.1%
9–11	18.0%	26.3%	29.6%	26.0%
≥12	16.0%	21.5%	25.8%	36.7%
Sex				
Male	22.5%	25.2%	22.5%	29.9%
Female	20.3%	26.1%	23.6%	30.1%
Maternal smoking during pregnancy				
No	20.5%	22.8%	23.7%	33.0%
Yes	23.0%	30.8%	21.9%	24.3%
Birthweight (grams)				
<2500	30.6%	27.8%	19.2%	22.4%
2500–2999	22.7%	26.1%	24.4%	26.8%
3000–3499	20.5%	26.4%	22.2%	30.9%
≥3500	19.2%	23.8%	24.0%	33.1%
Maternal prepregnancy body mass index				
Underweight	24.7%	26.9%	24.7%	23.7%
Normal	20.9%	25.9%	23.3%	29.9%
Overweight	21.3%	23.2%	23.8%	31.7%
Obese	28.2%	16.2%	23.2%	32.4%

Table 3 shows the association of the confounding variables with three outcomes (body mass index, lean mass index and visceral fat thickness). Body mass index was lower among subjects in the extreme categories of family income, whereas lean mass index and visceral fat thickness were negatively associated with family income. Body mass index, lean mass and visceral fat thickness were lower among those subjects whose mother had completed secondary school, whereas a positive association was observed for birthweight and maternal prepregnancy body mass index. Lean mass index and visceral fat thickness were higher among male subjects.

**Table 3:** Mean body mass index, lean mass index and visceral fat thickness according to confounding variables.

	Mean body mass index (kg/m^2^)	Mean lean mass index (kg/m^2^)	Mean visceral fat thickness (cm)
Family income at birth (quintiles)	P = 0.008*	P < 0.001*	P < 0.001*
1^st^	26.4	17.0	6.0
2^nd^	27.1	17.0	6.0
3^rd^	27.0	16.6	6.1
4^th^	27.3	16.7	5.5
5^th^	26.4	16.3	
Maternal schooling (years)	P = 0.007*	P < 0.001**	P < 0.001**
0–4	26.8	16.9	6.0
5–8	27.1	16.8	6.0
9–11	26.9	16.6	5.8
≥12	26.1	16.2	5.4
Sex	P = 0.10	P < 0.001	P < 0.001
Male	27.0	18.7	6.9
Female	26.7	14.8	4.9
Maternal smoking during pregnancy	P = 0.02	P = 0.008	P = 0.13
No	26.7	16.6	5.9
Yes	27.1	16.9	6.0
Birthweight (grams)	P < 0.001*	P < 0.001**	P = 0.003*
<2500	25.9	16.2	5.7
2500–2999	26.7	16.4	5.9
3000–3499	26.6	16.7	5.8
≥3500	27.5	17.1	6.1
Maternal prepregnancy body mass index	P < 0.001**	P < 0.001**	P < 0.001**
Underweight	24.8	16.3	5.6
Normal	26.4	16.6	5.8
Overweight	28.2	16.9	6.1
Obese	29.8	17.5	6.5

*Test of heterogeneity.

**Test of linear trend.

Neither body mass index nor the prevalence of overweight were associated with duration of breastfeeding (Table 4). Fat mass index at 30 years was lower among those subjects who had been breastfed for at least 6 months, but the confidence interval barely included the reference. On the other hand, lean mass index was positively associated with breastfeeding duration. Concerning abdominal fat compartments, visceral fat thickness was inversely associated with breastfeeding duration, even after controlling for possible confounding variables. In contrast, breastfeeding duration was not associated with subcutaneous abdominal fat thickness (Table 4).

**Table 4:** Association of body composition at 30 years with breastfeeding duration.

	Regression coefficient (95% confidence interval)	
	Crude	Adjusted^#^
**Body mass index (kg/m** ^**2**^ **)**		
Breastfeeding duration	P = 0.88*	P = 0.86*
<1 month	Reference (0)	Reference (0)
1–2.9 months	−0.13 (−0.68; 0.41)	0.06 (−0.48; 0.60)
3–5.9 months	0.06 (−0.49; 0.62)	0.17 (−0.39; 0.72)
≥6 months	−0.10 (−0.62; 0.43)	−0.05 (−0.58; 0.48)
**Prevalence ratio of overweight**		
Breastfeeding duration	P = 0.25*	P = 0.13*
<1 month	Reference (1)	Reference (1)
1 – 2.9 months	1.00 (0.92; 1.09)	1.03 (0.95; 1.12)
3–5.9 months	1.03 (0.95; 1.12)	1.05 (0.96; 1.14)
≥6 months	0.95 (0.87; 1.03)	0.96 (0.88; 1.04)
**Fat mass index (kg/m** ^**2**^ **)**		
Breastfeeding duration	P = 0.23*	P = 0.08*
<1 month	Reference (0)	Reference (0)
1–2.9 months	−0.04 (−0.47; 0.40)	−0.05 (−0.44; 0.33)
3–5.9 months	0.16 (−0.29; 0.60)	0.07 (−0.33; 0.46)
≥6 months	−0.27 (−0.69; 0.15)	−0.37 (−0.74; 0.01)
**Lean mass index (kg/m** ^**2**^ **)**		
Breastfeeding duration	P = 0.89*	P = 0.03**
<1 month	Reference (0)	Reference (0)
1–2.9 months	0.02 (−0.25; 0.28)	0.19 (0.01; 0.37)
3–5.9 months	0.08 (−0.19; 0.36)	0.24 (0.06; 0.43)
≥6 months	0.08 (−0.18; 0.34)	0.21 (0.04; 0.39)
**Waist circumference (cm)**		
Breastfeeding duration	P = 0.89*	P = 0.76*
<1 month	Reference (0)	Reference (0)
1–2.9 months	−0.44 (−1.69; 0.80)	0.26 (−0.90; 1.41)
3–5.9 months	−0.28 (−1.55; 1.01)	0.16 (−1.03; 1.36)
≥6 months	−0.62 (−1.82; 0.58)	−0.28 (−1.41; 0.84)
**Visceral fat thickness (cm)**		
Breastfeeding duration	P = 0.001**	P = 0.005**
<1 month	Reference (0)	Reference (0)
1–2.9 months	−0.14 (−0.35; 0.06)	−0.02 (−0.21; 0.16)
3–5.9 months	−0.25 (−0.47; −0.04)	−0.12 (−0.31; 0.07)
≥6 months	−0.31 (−0.51; −0.11)	−0.23 (−0.41; −0.05)
**Subcutaneous abdominal fat thickness (cm)**		
Breastfeeding duration	P = 0.44**	P = 0.19**
<1 month	Reference (0)	Reference (0)
1–2.9 months	−0.03 (−0.14; 0.08)	−0.03 (−0.13; 0.08)
3–5.9 months	−0.03 (−0.14; 0.09)	−0.04 (−0.15; 0.07)
≥6 months	−0.05 (−0.15; 0.06)	−0.07 (−0.17; 0.03)

*P-value for heterogeneity. **P-value for linear trend.

^#^Adjusted for: Family income at birth, parental schooling, household assets index, maternal smoking during pregnancy, gender, birthweight, maternal prepregnancy body mass index, maternal height, and skin color.

As was seen for total breastfeeding, duration of predominant breastfeeding was negatively associated with visceral fat thickness and positively related to lean mass index. No association was observed for the remaining body composition variables (Table 5).

**Table 5:** Association of body composition at 30 years with predominant breastfeeding duration.

	Regression coefficient (95% confidence interval)	
	Crude	Adjusted^#^
**Body mass index (kg/m** ^**2**^ **)**		
Predominant breastfeeding duration	P = 0.95*	P = 0.97*
<1 month	Reference (0)	Reference (0)
1–1.9 months	0.01 (−0.62; 0.64)	0.13 (−0.49; 0.75)
2–2.9 months	−0.14 (−0.69; 0.42)	0.02 (−0.53; 0.58)
≥3 months	0.02 (−0.46; 0.49)	0.09 (−0.38; 0.57)
**Prevalence ratio of overweight**		
Predominant breastfeeding duration	P = 0.91*	P = 0.93*
<1 month	Reference (1)	Reference (1)
1–1.9 months	1.01 (0.92; 1.12)	1.03 (0.94; 1.13)
2–2.9 months	0.98 (0.90; 1.07)	1.01 (0.93; 1.10)
≥3 months	1.01 (0.94; 1.08)	1.02 (0.95; 1.10)
**Fat mass index (kg/m** ^**2**^ **)**		
Predominant breastfeeding duration	P = 0.96*	P = 0.19*
<1 month	Reference (0)	Reference (0)
1–1.9 months	0.12 (−0.38; 0.62)	0.16 (−0.29; 0.60)
2–2.9 months	0.00 (−0.45; 0.44)	−0.23 (−0.62; 0.17)
≥3 months	0.02 (−0.36; 0.40)	−0.24 (−0.58; 0.10)
**Lean mass index (kg/m** ^**2**^ **)**		
Predominant breastfeeding duration	P = 0.58*	P = 0.002**
<1 month	Reference (0)	Reference (0)
1–1.9 months	0.06 (−0.25; 0.36)	0.16 (−0.04; 0.37)
2–2.9 months	−0.15 (−0.53; 0.12)	0.18 (0.00; 0.36)
≥3 months	−0.04 (−0.27; 0.20)	0.25 (0.10; 0.41)
**Waist circumference (cm)**		
Predominant breastfeeding duration	P = 0.11*	P = 0.70*
<1 month	Reference (0)	Reference (0)
1–1.9 months	0.13 (−1.30; 1.57)	0.49 (−0.85; 1.83)
2–2.9 months	−1.21 (−2.48; 0.06)	−0.21 (−1.39; 0.98)
≥3 months	−0.93 (−2.01; 0.15)	−0.26 (−1.28; 0.76)
**Visceral fat thickness (cm)**		
Predominant breastfeeding duration	P < 0.001**	P = 0.03**
<1 month	Reference (0)	Reference (0)
1–1.9 months	−0.12 (−0.36; 0.12)	−0.08 (−0.30; 013)
2–2.9 months	−0.39 (−0.60; −0.17)	−0.17 (−0.36; 0.02)
≥3 months	−0.34 (−0.52; −0.16)	−0.18 (−0.34; −0.01)
**Subcutaneous abdominal fat thickness (cm)**		
Predominant breastfeeding duration	P = 0.87*	P = 0.10**
<1 month	Reference (0)	Reference (0)
1–1.9 months	−0.02 (−0.15; 0.11)	−0.01 (−0.13; 0.11)
2–2.9 months	0.01 (−0.11; 0.12)	−0.02 (−0.13; 0.09)
≥3 months	−0.03 (−0.12; 0.06)	−0.08 (−0.17; 0.02)

*P-value for heterogeneity. **P-value for linear trend.

^#^Adjusted for: Family income at birth, parental schooling, household assets index, maternal smoking during pregnancy, gender, birthweight, maternal prepregnancy body mass index, maternal height, and skin color.

Table 6 shows that, among subjects breastfed for less than one month, all outcomes showed monotonically increasing values with additional copies of the A allele in the FTO genotype (rs9939609). Associations among subjects who were breastfed for one month or longer tended to be in the same direction but showed lower magnitude and were less consistent; for all outcomes, heterozygous subjects had values of the outcomes that included the zero value, that is, were not significantly different from the TT genotype. For all outcomes except fat mass index, the AA and TT genotypes were significantly different. Interactions had p values of 0.05 or less for body mass index, fat mass index and waist circumference, and values between 0.05 and 0.15 for overweight prevalence, visceral and abdominal fat thickness.

**Table 6:** Association of body composition at 30 years with breastfeeding duration, according to FTO genotype (rs9939609).

	Adjusted regression coefficient (95% confidence interval)^#^		P-value - interaction
	Breastfeeding duration in months		
	<1	≥1	
	**Body mass index (kg/m** ^**2**^ **)**		
FTO genotype	p < 0.001**	P = 0.005*	0.02
TT	Reference (0)	Reference (0)	
TA	1.14 (0.13; 2.15)	−0.01 (−0.55; 0.53)	
AA	2.58 (1.30; 3.87)	1.05 (0.34; 1.75)	
	**Prevalence ratio of overweight**		
FTO genotype	P = 0.004**	P = 0.05**	0.08
TT	Reference (1)	Reference (1)	
TA	1.17 (0.98; 1.40)	1.02 (0.94; 1.11)	
AA	1.33 (1.09; 1.62)	1.11 (1.01; 1.23)	
**Fat mass index (kg/m** ^**2**^ **)**			
FTO genotype	P = 0.009**	P = 0.08*	0.05
TT	Reference (0)	Reference (0)	
TA	0.63 (−0.10; 1.36)	−0.16 (−0.54; 0.22)	
AA	1.25 (0.30; 2.19)	0.38 (−0.12; 0.89)	
	**Lean mass index (kg/m** ^**2**^ **)**		
FTO genotype	P = 0.02**	P = 0.008**	0.41
TT	Reference (0)	Reference (0)	
TA	0.36 (0.04; 0.68)	0.13 (−0.05; 0.31)	
AA	0.44 (0.02; 0.86)	0.32 (0.09; 0.56)	
	**Waist circumference (cm)**		
FTO genotype	P < 0.001**	P = 0.005*	0.01
TT	Reference (0)	Reference (0)	
TA	2.21(0.04; 4.37)	−0.32 (−1.48; 0.83)	
AA	5.65 (2.88; 8.41)	2.04 (0.53; 3;55)	
	**Visceral fat thickness (cm)**		
FTO genotype	P = 0.01**	P = 0.07**	0.13
TT	Reference (0)	Reference (0)	
TA	0.26 (−0.09; 0.61)	0.03 (−0.14; 0.21)	
AA	0.57 (0.13; 1.02)	0.23 (0.00; 0.46)	
	**Subcutaneous abdominal fat thickness (cm)**		
FTO genotype	P = 0.003**	P = 0.002*	0.11
TT	Reference (0)	Reference (0)	
TA	0.10 (−0.10; 0.31)	−0.01 (−0.11; 0.10)	
AA	0.42 (0.16; 0.68)	0.21 (0.08; 0.35)	

*P-value for heterogeneity. **P-value for linear trend.

^#^Adjusted for: Family income at birth, parental schooling, household assets index, maternal smoking during pregnancy, gender, birthweight, maternal prepregnancy body mass index, maternal height, and skin color.

Table 7 shows that breastfeeding for longer durations was not associated with a further attenuation of the association of the rare allele with the outcomes.

**Table 7:** Association of body composition at 30 years with breastfeeding duration, according to FTO genotype (rs9939609).

	Adjusted regression coefficient (95% confidence interval)^#^				P-value - interaction
	Breastfeeding duration in months				
	<1	1–2.9	3–5.9	≥6	
	**Body mass index (kg/m** ^**2**^ **)**				
FTO genotype	p < 0.001**	p = 0.09*	p = 0.12**	P = 0.18*	0.07
TT	Reference (0)	Reference (0)	Reference (0)	Reference (0)	
TA	1.14 (0.13; 2.15)	−0.11 (−1.01; 0.78)	0.19 (−0.81; 1.20)	−0.13 (−1.05; 0.78)	
AA	2.58 (1.30; 3.87)	1.06 (−0.09; 2.20)	1.08 (−0.20; 2.37)	0.98 (−0.27; 2.23)	
	**Prevalence ratio of overweight**				
FTO genotype	P = 0.004**	P = 0.07**	P = 0.05**	P = 0.87*	0.03
TT	Reference (1)	Reference (1)	Reference (1)	Reference (1)	
TA	1.17 (0.98; 1.40)	1.02 (0.88; 1.19)	1.06 (0.92; 1.23)	0.97 (0.84; 1.11)	
AA	1.33 (1.09; 1.62)	1.19 (1.01; 1.40)	1.19 (1.01; 1.41)	0.97 (0.81; 1.16)	
	**Fat mass index (kg/m** ^**2**^ **)**				
FTO genotype	P = 0.009**	P = 0.28*	P = 0.09*	P = 0.93*	0.07
TT	Reference (0)	Reference (0)	Reference (0)	Reference (0)	
TA	0.63 (−0.10; 1.36)	−0.25 (−0.90; 0.40)	−0.17 (−0.88; 0.55)	−0.12 (−0.75; 0.51)	
AA	1.25 (0.30; 2.19)	0.38 (−0.45; 1.22)	0.77 (−0.14; 1.68)	−0.09 (−0.96; 0.78)	
	**Lean mass index (kg/m** ^**2**^ **)**				
FTO genotype	P = 0.02**	P = 0.14**	P = 0.03**	P = 0.19**	0.54
TT	Reference (0)	Reference (0)	Reference (0)	Reference (0)	
TA	0.36 (0.04; 0.68)	0.16 (−0.14; 0.47)	0.23 (−0.11; 0.56)	0.01 (−0.29; 0.31)	
AA	0.44 (0.02; 0.86)	0.28 (-0.11; 0.68)	0.36 (−0.06; 0.79)	0.33 (−0.08; 0.75)	
	**Waist circumference (cm)**				
FTO genotype	P < 0.001**	P = 0.10*	P = 0.11*	P = 0.26*	0.04
TT	Reference (0)	Reference (0)	Reference (0)	Reference (0)	
TA	2.21(0.04; 4.37)	−0.57 (−2.51; 1.36)	-0.08 (−2.03; 2.19)	−0.53 (-2.50; 1.43)	
AA	5.65 (2.88; 8.41)	1.94 (−0.53; 4.41)	2.48 (−0.23; 5.18)	1.59 (−1.07; 4.25)	
	**Visceral fat thickness (cm)**				
FTO genotype	P = 0.01**	P = 0.13**	P = 0.38*	P = 0.11**	0.29
TT	Reference (0)	Reference (0)	Reference (0)	Reference (0)	
TA	0.26 (−0.09; 0.61)	0.11 (−0.20; 0.42)	−0.27 (−0.60; 0.05)	0.15 (−0.13; 0.44)	
AA	0.57 (0.13; 1.02)	0.32 (−0.08; 0.71)	−0.03 (−0.45; 0.39)	0.29 (−0.09; 0.68)	
	**Subcutaneous abdominal fat thickness (cm)**				
FTO genotype	P = 0.003**	P = 0.04*	P = 0.02**	P = 0.35*	0.22
TT	Reference (0)	Reference (0)	Reference (0)	Reference (0)	
TA	0.10 (−0.10; 0.31)	−0.09 (−0.26; 0.08)	0.09 (−0.11; 0.28)	0.00 (−0.17; 0.17)	
AA	0.42 (0.16; 0.68)	0.18 (−0.04; 0.40)	0.31 (0.07; 0.56)	0.15 (−0.07; 0.38)	

*P-value for heterogeneity. **P-value for linear trend.

^#^Adjusted for: Family income at birth, parental schooling, household assets index, maternal smoking during pregnancy, gender, birthweight, maternal prepregnancy body mass index, maternal height, and skin color.

## Discussion

In our study, duration of breastfeeding was not associated with body mass index or the risk of being overweight at 30 years. This is in contrast to a recent meta-analysis that reported that breastfeeding is associated with a 13% decrease in the odds of overweight/obesity in high-quality studies^6^. Our study complied with the definition of high-quality studies used in that review. On the other hand, breastfeeding was associated with a small decrease in the thickness of visceral fat layer and an increase in lean mass index. Such associations may have long-term impact on the development of metabolic diseases, given their association with the development of metabolic disorders^26^.

Interestingly, we observed that breastfeeding duration moderated the association of a genetic variant in the *FTO* gene with adiposity in adulthood. The only two published studies on this topic used BMI as the only outcome and were restricted to children and young adolescents^11,12^. Ours is the first study to investigate this interaction in adults, and also to report on body composition outcomes other than BMI.

The present results are unlikely to have been due to selection bias, because follow-up rate was independent of breastfeeding duration. Moreover, estimates were adjusted for several possible confounding variables that were assessed in early childhood, reducing the likelihood of recall bias and of poor control for confounding. Conversely to studies from high-income countries^24^, residual confounding due to imperfect adjustment for socioeconomic status is unlikely in our population, where there is no strong social patterning of breastfeeding duration. In fact, the proportion of subjects who were breastfed at 6 months was higher in the extreme categories of family income, whereas lean mass and visceral fat thickness were negatively associated with socioeconomic status. Moreover, when residual confounding occurs, associations tend to be weakened after adjustment, rather than remaining virtually unchanged, as we observed in our study. These small differences between crude and adjusted estimates are not due to poor measurement of confounders, since the estimates were adjusted for several socioeconomic and demographic variables that were measured with a short-term recall, therefore, measurement error is unlikely. Moreover, we observed in our cohort that low socioeconomic status in childhood was associated with increased risk of mortality in our cohort^27^. If socioeconomic status had been poorly measured, we would not have observed such association.

This study has some limitations. Huttly *et al*.^28^ carried out a validation study on the information on breastfeeding duration, and observed that about one of each four mothers misclassified the information on breastfeeding duration, but in most of the cases to a nearest category. Such classification error would tend to underestimate the magnitude of the associations of breastfeeding with body composition. Therefore, the observed associations of breastfeeding with body composition and the moderation of the association between the *FTO* variant rs9939609 and body composition should not be considered as due to this measurement error. If breastfeeding duration had been measured more precisely, it is likely stronger associations would be observed.

Because we tested for the association of breastfeeding with seven different outcomes, the observed associations might have occurred by chance due to inflations in type-1 error. However, it is important to consider that these outcomes are correlated with one another, so the inflation is smaller than one would expect based on the number of outcomes alone^29^. Moreover, the association of breastfeeding with lean mass index and visceral fat thickness was replicated for predominant breastfeeding, associations that did not achieve conventional levels of statistical significance were mostly consistent with significant associations and the observed number of associations that achieved conventional levels of statistical significance was higher than what would be expected by chance alone.

With respect to the association of breastfeeding with specific body composition measurements, Durmus *et al*.^22^ also observed that breastfeeding was negatively associated with general and abdominal fat measures, but not with body mass index at 6 years of age. This issue should be taken into consideration in the design of new studies aimed at assessing the relationship of infant feeding with body composition.

Concerning the biological plausibility of the association between breastfeeding and body composition, it has been proposed that breast milk would modulate infant gut microbiota colonization and development, e.g., higher counts of Bifidobacteria have been reported among breastfed infants^30–32^. Differences in diet could be another mechanism, with children who had been breasted being more likely to have healthy dietary patterns as adults^33,34^. Moreover, children who had been breastfed would have a higher level of satiety^8^. The finding that breastfeeding moderates the adipogenic effect of the rs9939609 variant allele has also been observed in other settings^11,12^, reinforcing the notion that breastfeeding may program the development of adiposity in later life, because the *FTO* gene is associated with food intake^9,10^.

There was stronger statistical evidence supporting an interaction between the rs9939609 variant and breastfeeding when the latter was categorised as <1 month vs. ≥1 month, compared to the more detailed categorisation of <1, 1–2.9; 3–5.9 and ≥6 months. Moreover, the association was not stronger than that already achieved in the category of 1–2.9 months of breastfeeding duration. In the absence of clear differences, it was possible to combine the latter three groups into a single one to obtain more precise estimates. This finding is in accordance with the notion of nutritional adequacy hypothesis – i.e., upon achieving the required duration of breastfeeding to moderate the association of the rs9939609 variant with adiposity, further extending breastfeeding duration does not exert any additional benefit on this regard. This hypothesis has been proposed in other Gene × Environment interaction analyses involving breastfeeding, genetic variants in the *FADS2* gene and intelligence^35^.

Our findings suggest that breastfeeding has long-term consequences on body composition, breastfeeding was negatively associated with the thickness of visceral fat layer and positively with lean mass index at 30 years of age. Furthermore, it seems that breastfeeding moderates the association of *FTO* genotype with body composition.

## Methods

### Participants

In 1982, the five maternity hospitals in Pelotas, a southern Brazilian city, were visited daily and all births were identified. The 5914 livebirths whose family lived in the urban area of the city were examined and their mothers interviewed soon after delivery, and these subjects have been followed up on several occasions. In 1984 (mean age of 19 months) and 1986 (mean age of 42 months), the households located in the urban area of the city were visited, and 87% and 84% of the original cohort was identified. The children were examined and the mothers interviewed using a standardized questionnaire. From June 2012 to February 2013, cohort members were invited to visit the research clinic to be interviewed and examined. Further details on the study methodology have been published elsewhere^36,37^.

### Ethics Statement

The Ethical Review Board of the Faculty of Medicine of the Federal University of Pelotas approved the study, and we obtained written informed consent from all participants. All methods were performed in accordance with relevant guidelines and regulations.

### Exposure

Information on breastfeeding duration was collected in the 1984 and 1986 visit and the earliest information on the age at which breastfeeding stopped completely was used. Data on the age of introduction of complementary foods was also collected in childhood, and duration of predominant breastfeeding assessed the age at which foods other than breast milk, teas or water were introduced^38^.

In 2004–05, we tried to follow the whole cohort, and participants were interviewed and donated a blood sample. DNA was extracted and genotyped using the Illumina HumanOmni2.5-8v1 array. Individual samples were excluded on the basis of sex mismatches (only for non-pseudoautosomal X-chromosome SNPs; heterozygosity threshold: 0.02), minimal or excessive heterozygosis (outside the range of median ±1.5*IQR of heterozygosity rate), disproportionate levels of individual missingness (>3%) and cryptic relatedness (>0.1 kinship)^39^. The *FTO* SNP rs9939609 was imputed (INFO metric of imputation quality >0.99^40^) as A-allele dosages (with T being the non-effect allele). Imputation was performed by pre-phasing using SHAPEIT^41^ and the actual imputation using IMPUTE2^42,43^. Hardy-Weinberg equilibrium was assessed using X^2^ test.

### Outcomes

Body mass index was estimated from weight and height measurements. Weight was measured using the Bod POD® scale and height a portable stadiometer (aluminum and wood) with accuracy of 0.1 cm. Overweight was defined by a body mass index of 25 kg/m^2^ or more.

Body composition was evaluated with dual-energy x-ray absorptiometry (DXA Lunar Prodigy), and fat mass and lean mass index in kg/m^2^ were estimated.

Visceral and subcutaneous abdominal fat thickness were estimated using a Toshiba Xario (Toshiba Medical Systems Corp., Tokyo, Japan) ultrasound with a 3.5-MHZ convex probe, according to validated protocols^44^. Visceral fat thickness was defined by the distance between the peritoneum and lumbar spine at the intersection between the xyphoid line and the waist circumference. Subcutaneous abdominal fat thickness was estimated by the distance between the posterior line of dermis and the outer bowel wall. Both measurements were taken from static images at the end of a quiet expiration. Pregnant women or up to 3-month post-partum were excluded from this assessment.

Waist circumference was measured twice, after a gentle expiration, using a flexible tape (Cescorf®, Porto Alegre, Brazil) with an accuracy of 0.1 cm at the narrowest part of the trunk, identified as the midpoint between the lowest rib margin and the iliac crest. If the difference between the measurements was greater than 1 cm, two additional measures were taken.

### Confounders

The following variables measured in the perinatal study were considered as possible confounders: family income, maternal schooling, household assets index (estimated through factor analysis and based in the ownership of household goods, evaluated in the 1984 and 1986 visits), maternal smoking during pregnancy, maternal prepregnancy body mass index (information on prepregnancy weight was gathered from antenatal care records or by maternal recall), maternal height (measured by the research team), skin color and birthweight (recorded by the hospital staff using calibrated pediatric scales). Information on family income in minimum wage was gathered in five categories. In order to classify the subjects into quintiles of family income, a principal component analysis using four variables (delivery payment mode, maternal schooling, height and skin color) was carried out. A score based in the first component was used to rank the individuals within family income categories, and cut-off points were obtained within each income group so that five nearly equal size groups were created^45^. Genomic ancestry analysis was based on 370539 SNPs shared by samples from the HapMap Project, the Human Genome Diversity Project (HGDP), and the Pelotas cohort. The following HapMap samples were used as external panels: 266 Africans, 262 Europeans (American and Italian), 77 admixed Mexican Americans, 83 African Americans, and 93 Native Americans from the HGDP. For each individual, the proportion of European, African American, and Native American ancestry was estimated^46^.

### Statistical Analyses

Statistical analyses were performed using Stata version 13 (StataCorp, College Station, TX, USA). Initially, the analyses were stratified by sex, but we presented the pooled results because there was no statistical evidence of interaction (p-values for interaction >0.1). Means were compared using ANOVA, and the chi-square test was used to compare proportions. Statistical comparisons between groups were based on tests of heterogeneity and linear trend, and we presented the one with the lower p-value. Multivariable linear regression was used to adjust the estimates for confounders. In the linear regression, we graphically tested the normality and homoscedasticity (homogeneity of variance) of residuals. Because the odds ratio overestimates the prevalence ratio, and this overestimation increases as the prevalence of the outcome increases, Poisson regression with robust adjustment of the variance was used to estimate the prevalence ratio of overweight^47^. We also evaluated whether the association between the *FTO* variant (rs9939609) and body composition variables was modified by breastfeeding duration. To properly adjust for confounding effects of genomic ancestry on the Gene × Environment interaction (*FTO* × Breastfeeding) term, not only a main effect term for genomic ancestry was included, but also Ancestry × *FTO* and Ancestry × Breastfeeding terms^48^. To incorporate this methodology while avoiding estimating too many parameters in the model, only African ancestry was adjusted for in this way given the importance of population substructure for genetic associations^49^.

### Data availability

The dataset analysed during the current study are available from the corresponding author on reasonable request.
